# Psychiatric care in university population

**DOI:** 10.1192/j.eurpsy.2021.1931

**Published:** 2021-08-13

**Authors:** N.M. Casado Espada, A. Flores, M.T. Lozano López, B. Bote Bonaechea, A.B. Sánchez García, M. Rodríguez, C. Roncero

**Affiliations:** 1 Psychiatry, University of Salamanca Healthcare Complex. Institute of Biomedicine of Salamanca. Psychiatric Area, University of Salamanca, Salamanca, Spain; 2 Psychiatry, University of Salamanca, Salamanca, Spain; 3 Psychiatry, University of Salamanca Healthcare Complex. Institute of Biomedicine of Salamanca, Salamanca, Spain; 4 Social Affairs Service, University of Salamanca, Salamanca, Spain; 5 Psychiatry, Psychiatry, University of Salamanca Healthcare Complex. Institute of Biomedicine of Salamanca. Psychiatric Area, University of Salamanca, Salamanca, Spain, Salamanca, Spain

**Keywords:** university population, mental health care, mental health prevention, mental health promotion

## Abstract

**Introduction:**

It is well-known that university students experience high levels of mental health problems (1). University life presents changes and challenges that can be stressful and may affect the mental health of its community (2,3). More than 20 years ago, the Social Affairs Service (SAS) of the University of Salamanca started a program that ensured the mental health care in their community. The Psychiatric Care Unit is part of this program and its objectives are: 1) to detect serious mental disorders; 2) treat mild mental disorders; 3) give information to prevent illness and promote mental health; 4) serve as support in patients with previous follow-up that has been discontinued due to the beginning of their studies; 5) liaise with referral psychiatrists.

**Objectives:**

To make known a Psychiatric Care Unit targeted in the university community

**Methods:**

18 people between 19 and 52 years old (22% male, 78% female) were evaluated between November and December of 2020 in the Psychiatric Care Unit of the Social Affairs Service (PCU-SAS, University of Salamanca). The assessment consisted in an interview carried out by a psychiatrist, in the presence of a medical graduate. Every patient belong to the university community (students/ staff).

**Results:**

The most frequent diagnosis in the sample is Adjustment Disorder (F43.2). Substance use, eating disorders, low-self-concept, perfectionism and emotional dysregulation are very prevalent symptoms along our sample.

**Conclusions:**

Universities should invest in creating environments that promote student and staff mental wellbeing. However, the current body of evidence is scarce and more research is needed to recommend what are the best strategies(4).

**Disclosure:**

No significant relationships.

